# Evaluation of a self-administered version of the Revised Amyotrophic Lateral Sclerosis Functional Rating Scale (ALSFRS-R)

**DOI:** 10.1055/s-0046-1827063

**Published:** 2026-09-08

**Authors:** Thais Alves Cunha, Laura Carvalheira Dourado, Glauciane Costa Santana, Mário Teruo Yanagiura, Leonardo Judson Galvão-Lima, Sancha Helena de Lima Vale, José Brandão-Neto, Mário Emílio Teixeira Dourado-Junior, Lucia Leite-Lais

**Affiliations:** 1 Universidade Federal do Rio Grande do Norte, Programa de Pós-graduação em Ciências da Saúde, Natal RN, Brazil.; 2 Universidade Federal do Rio Grande do Norte, Programa de Pós-graduação em Nutrição, Natal RN, Brazil.; 3 Universidade Federal do Rio Grande do Norte, Hospital Universitário Onofre Lopes, Natal RN, Brazil.; 4 Universidade Federal do Rio Grande do Norte, Laboratório de Inovação Tecnológica em Saúde, Natal RN, Brazil.; 5 Universidade Federal do Rio Grande do Norte, Departamento de Medicina Integrada, Natal RN, Brazil.; 6 Universidade Federal do Rio Grande do Norte, Departamento de Nutrição, Natal RN, Brazil.

**Keywords:** Amyotrophic Lateral Sclerosis, Self-Assessment, Remote Patient Monitoring

## Abstract

**Background:**

The Revised Amyotrophic Lateral Sclerosis Functional Rating Scale (ALSFRS-R) is a standard tool for evaluating functional decline in patients with ALS. Despite its clinical value, administration by healthcare professionals can be time-consuming and resource intensive.

**Objective:**

To assess the reliability and feasibility of a self-administration version of the ALSFRS-R as an alternative for use in clinical and research settings.

**Methods:**

The present was an observational, analytical, prospective, single-center study involving ALS patients followed at the Neurology Outpatient Clinic of the Hospital Universitário Onofre Lopes (HUOL). Three independent assessments of the ALSFRS-R were conducted: one self-administered version and two interviewer-administered versions performed by different researchers during face-to-face consultations. Interrater reliability was assessed using intraclass correlation coefficients (ICCs), and agreement among the three versions was analyzed using Bland-Altman plots.

**Results:**

A total of 43 participants were included in the study, with a mean age of 57 ± 11.67 years. The ICCs indicated high reliability for both the total ALSFRS-R score and its functional domains. Linear regression analyses demonstrated strong agreement between the two researchers (R ^2^ = 0.98, *p* < 0.001), as well as between each researcher and the self-administered version (R ^2^ = 0.90 and 0.88, respectively). Bland-Altman analyses showed minimal bias and acceptable limits of agreement across all comparisons.

**Conclusion:**

The self-administered version of the ALSFRS-R demonstrated high reliability and strong agreement with the researcher-administered versions, supporting its potential use for remote monitoring of ALS patients. Nonetheless, it should not replace professional assessments in clinical trial settings. Further research is warranted to validate its applicability in broader clinical contexts and diverse patient populations.

## INTRODUCTION

Amyotrophic lateral sclerosis (ALS) is a progressive neurodegenerative disease affecting both upper and lower motor neurons, 1 leading to bulbar paralysis, limb muscle atrophy, and ultimately death due to dysphagia or respiratory muscle paralysis. The average survival time after diagnosis ranges from 2 to 4 years. 2

Disease severity and functional decline in ALS are commonly assessed using the Revised Amyotrophic Lateral Sclerosis Functional Rating Scale (ALSFRS-R). It consists of 12 items covering four functional domains: bulbar, fine motor, gross motor, and respiratory. It can be administered by healthcare professionals in person, via telephone, or online. The ALSFRS-R is a widely used, accessible, and multidimensional tool that is sensitive to clinical changes and is recognized as a meaningful outcome measure in routine clinical practice. 3 4 5

Assessing these functions is essential in both clinical practice and research. 1 However, data collection in ALS presents significant challenges, as in-person visits often require patients to travel to specialized centers. This process can be time-consuming, costly, and exhausting, as well as physically and emotionally taxing, particularly as the disease progresses. 4

This condition requires constant and comprehensive care. However, clinical follow-up typically depends on regular visits to specialized centers, which may involve long-distance travel and significant mobility challenges. These barriers often result in gaps in monitoring between appointments. Therefore, it is crucial to explore tools that allow for remote assessment and enable more flexible care, particularly for patients who live far from specialized services. 6

The ALSFRS-R does not require physical examinations or specialized equipment and has demonstrated high inter- and intrarater reliability. As such, self-administration at home allows for patient monitoring without the need for travel or scheduled appointments, offering greater flexibility in its application. 7

Patients have reported that self-monitoring provides a greater sense of control over the disease, while also allowing clinical consultations to become more flexible and better tailored to their individual needs. 8

A self-administration version of the ALSFRS-R can serve as a complement to in-person visits, supporting shared decision-making between healthcare professionals and patients. 3 Accordingly, the objective of this study was to evaluate the reliability of a self-administered version of the ALSFRS-R in patients with ALS.

## METHODS

### Study design and sample

This was a single-center, observational, analytical, and prospective study. It included patients with a confirmed diagnosis of ALS, as determined by a specialist, who were regularly followed up at the Neurology outpatient clinic of the Hospital Universitário Onofre Lopes (HUOL), Natal, Rio Grande do Norte, Brazil.

Patients were eligible regardless of their disease stage, provided they attended their routine consultation during the study period. Those who failed to complete the self-administered form within the week of their schedule consultation were excluded to minimize potential bias related to disease progression or response recall. Three independent assessments of the ALSFRS-R were compared:

- The self-administered version (self-adm): completed by the patient within the week of their consultation.
- The original version administered by researcher 1 (OVR1): conducted during the in-person consultation.
- The original version administered by researcher 2 (OVR2): also conducted during the same in-person consultation.

### Instrument and data collection

The original version of ALSFRS-R consists of 12 items that assess functional activities (speech, sialorrhea, swallowing, handwriting, food handling, hygiene and bathing, turning in bed and adjusting sheets, walking, climbing stairs, dyspnea, orthopnea, and respiratory insufficiency). It was developed for longitudinal monitoring of ALS patients. Each item is scored on a 5-point scale (0–4), where 0 indicates inability to perform the function and 4 represents normal function. The maximum total score is 48 points. 9

The self-administered version of the ALSFRS-R was adapted at HUOL's Neurology Outpatient Clinic, with clearer instructions and simplified multiple-choice statements to facilitate comprehension and accurate response selection ( **Supplementary Material** – available at https://www.arquivosdeneuropsiquiatria.org/wp-content/uploads/2026/05/ANP-2025.0488-Supplementary-Material.pdf ). It can be completed by the patient, with or without assistance, or by the caregiver.

**Figure 1:**
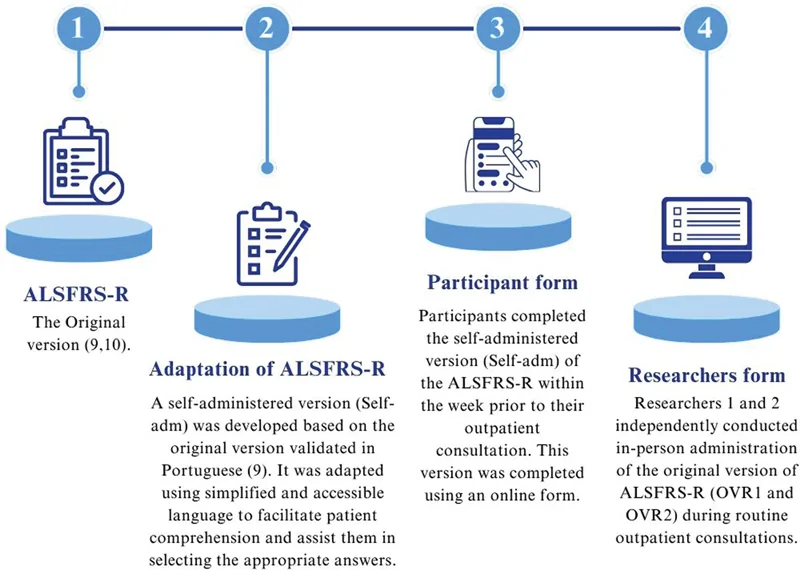
Flowchart of the adaptation and application of the Revised Amyotrophic Lateral Sclerosis Functional Rating Scale (ALSFRS-R) versions in the study protocol.

This study was approved by the Research Ethics Committee of HUOL/UFRN (no. 6.099.304), and all participants provided written informed consent. Data were collected between June 2023 and September 2024. The week prior to their outpatient consultation, patients completed the self-administered version of the ALSFRS-R via an online form. During the routine consultation, two researchers independently applied the original version of the scale. The data collection process is summarized in the flowchart presented in Figure 1 .

### Anthropometric assessment

The anthropometric assessment was performed using patients' Body Mass Index (BMI), calculated by weight (kg) divided by height (m ^2^ ). Body weight (kg) was measured using a calibrated digital scale with a maximum capacity of 500 kg (KN P/R 500/50, KN Waagen), and height (cm) was reported by the patient.

### Statistical analysis

Statistical analyses were performed using IBM SPSS Statistics for Windows (IBM Corp.) version 28, with a significance level set at 5% for all tests. The Kolmogorov-Smirnov test was used to assess the normality of quantitative variables. Parametric data were presented as mean and standard deviations (SD), whereas nonparametric data were reported as medians and interquartile ranges (IQR: Q1–Q3). Qualitative variables were presented as relative frequencies.

To assess interrater reliability between patients and researchers, intraclass correlation coefficients (ICCs) were calculated for both the total score and the domains scores of the self-administered version of the ALSFRS-R. The ICCs were derived using a two-way random-effects analysis of variance (ANOVA) model based on absolute agreement. An ICC greater than 0.70 was considered acceptable for group-level comparisons, while values above 0.90 were deemed suitable for individual-level assessments. 3 5 Paired t-tests were used to examine differences in mean scores between raters and across time points.

The Bland-Altman 10 method was used to evaluate the level of agreement between the three versions of the scale administration:

- Comparison 1: Self-administered version (Self-adm) versus original versions administered by Researcher 1 (OVR1).
- Comparison 2: Self-administered versus original versions administered by Researcher 2 (OVR2).
- Comparison 3: OVR1 versus OVR2 assessments.

A Bland-Altman plot was generated for each comparison. The x-axis represents the mean of both measurements, while the y-axis shows the difference between them. The central line indicates the mean difference, and the limits of agreement define the range within which most differences between measurements are expected to lie. 10

## RESULTS

The study included 43 participants, comprising 21 men (49%) and 22 women (51%). The mean age was 57 (11.67) years. The average disease duration was 30 (22–71) months, calculated from symptom onset to the date of scale completion. Most participants presented spinal-onset symptoms. Regarding response sources, 74% of the assessments were completed by the patients themselves, either independently or with assistance, while 26% were completed by caregivers. Participant characteristics are summarized in Table 1 .

**Table 1:** Clinical and nutritional characterization of participants

Variables		Descriptive statistics
**Sex, n (%)**	Male	21 (49)
	Female	22 (51)
**Mean age, years (SD)**		57 (11.67)
**Mean height, cm (SD)**		162 (8.86)
**Mean weight, kg (SD)**		67 (14.34)
** Mean BMI, kg/m ^2^ (SD) **		25.47 (5.24)
**Median disease time, months (Q1–Q3)**		30 (22–71)
**Noninvasive ventilation, n (%)**		11 (26)
**Site of onset, n (%)**	Spinal	31 (72)
	Bulbar	12 (28)
**Answer, n (%)**	Patient	12 (28)
	Patient with assistance	20 (46)
	Caregiver	11 (26)

Abbreviations: BMI, Body Mass Index; Q1, interquartile range 1; Q3, interquartile range 3; SD, standard deviation.

**Figure 2:**
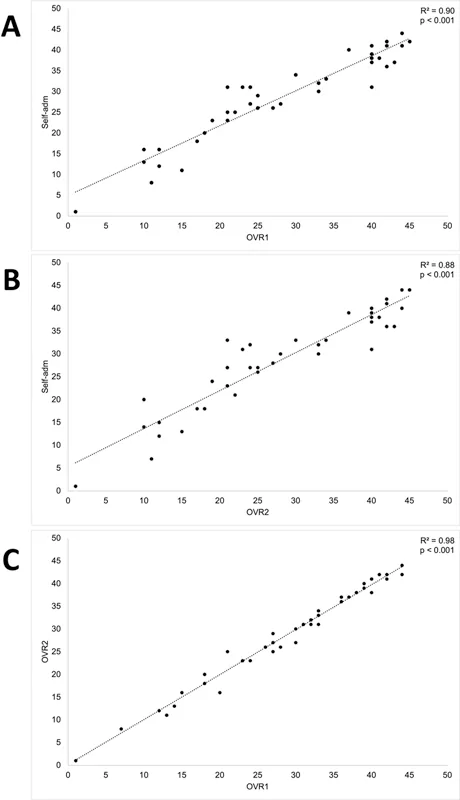
Linear regression analysis of ALSFRS-R scores. ( **A** ) Self-administered version (self-adm) vs. original version administered by researcher 1 (OVR1). ( **B** ) Self-administered vs. original version administered by researcher 2 (OVR2). ( **C** ) OVR1 vs. OVR2.

The ICC indicated that the total scale score demonstrated high reliability ( Table 2 ). Linear regression analysis ( Figure 2 ) showed excellent agreement between the two researchers, with R ^2^ = 0.98 ( *p* < 0.001). The agreement between researcher 1 (OVR1 and the patient yielded an R ^2^ of 0.90 ( *p* < 0.001), and between researcher 2 (OVR2 and the patient yielded an R ^2^ of 0.88 ( *p* < 0.001), indicating strong applicability of the scale.

**Table 2:** The ICCs for total and domain scores of the ALSFRS-R

ALSFRS-R	ICC	*p* -value
Total	0.95	*< 0.001*
Bulbar function	0.95	*< 0.001*
Fine motor function	0.93	*< 0.001*
Gross motor function	0.93	*< 0.001*
Respiratory function	0.85	*< 0.001*

Abbreviations: ALSFRS-R, Revised Amyotrophic Lateral Sclerosis Functional Rating Scale; ICC, intraclass correlation coefficient.

**Figure 3:**
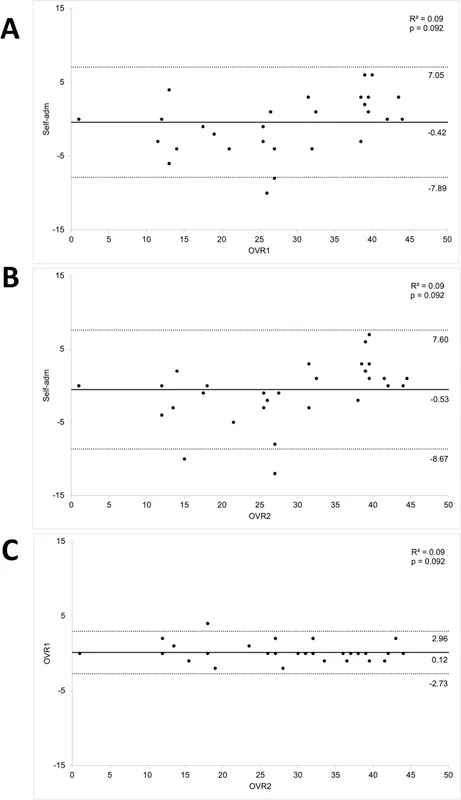
Bland-Altman plots comparing ALSFRS-R scores. ( **A** ) Self-administered version (aelf-adm) vs. original version administered by researcher 1 (OVR1). ( **B** ) Self-administered vs. original version administered by researcher 2 (OVR2). ( **C** ) OVR1 vs. OVR2.

Bland-Altman plots were constructed ( Figure 3 ) to assess agreement between the different methods of ALSFRS-R administration. The analyses revealed the following mean bias: self-adm versus OVR1: −0.4 (95% CI: −7.89–7.05); self-adm versus OVR2: −0.5 (95% CI: −8.67–7.60); and OVR1 versus OVR2: 0.1 (95% CI: −2.73–2.96). These findings indicate strong agreement across administration methods, with relatively narrow limits of agreement, supporting the reliability and practical applicability of the self-administered version.

## DISCUSSION

This study assessed the reliability of the self-administered version of the ALSFRS-R, demonstrating high reproducibility and strong agreement between researchers and ALS patients. The ICC indicated excellent reliability for the total score, while linear regression analyses revealed strong agreement not only between the researchers but also between each researcher and the patients. These results support the feasibility and reliability of the self-administered version for monitoring functional progression in ALS.

The self-administered version of the ALSFRS-R provides a practical alternative for monitoring disease progression outside the clinical setting. This is particularly valuable given that many patients must travel long distances to specialized centers for routine follow-ups. Facilitating at-home assessments allows for more flexible and individualized care, enabling clinic visits to be scheduled based on patient-specific needs. This approach not only reduces patients' physical and emotional burden but also promotes more efficient use of healthcare resources.

In previous studies, patients reported that self-monitoring offered a greater sense of control over their condition and allowed for more flexible clinical follow-up tailored to individual needs. Additionally, training patients and caregivers to use simple self-assessment tools proved to be feasible and generated reliable data, thereby reducing the necessity for travel to clinical centers. These findings highlight the importance of remote monitoring strategies in enhancing care delivery, particularly for geographically dispersed populations. 8 11 12

In our study, the strong agreement between patients and healthcare professionals further supports the reliability of self-assessment and highlights its potential to enhance patient autonomy in disease monitoring. It is important to note that a proportion of the assessments were completed by caregivers, reflecting real-world clinical conditions in ALS, particularly in more advanced stages. While this may introduce variability, it also enhances the representativeness of the sample and should be considered when interpreting the findings.

Despite its advantages, it is important to acknowledge the limitations of the ALSFRS-R. As an ordinal scale without linear weighting, score changes may reflect minor or substantial functional variations, depending on the specific item and response. This characteristic can reduce scale's sensitivity in detecting meaningful functional changes. Additionally, scores may improve with symptomatic treatment even as the disease progresses 13 and the scale may be less sensitive in more advanced stages. 7 These limitations underscore the need for cautious interpretation of ALSFRS-R results, particularly in clinical decision-making or in research contexts.

Additionally, we did not assess the correlation between ALSFRS-R scores and disease staging systems (e.g., MiToS or King's College staging), which could provide further insight into the clinical validity of the instrument and should be explored in future studies.

The ALSFRS-R was originally developed for administration by trained healthcare professionals in clinical trials settings, but its use has expanded to include an untrained personnel and patient self-administration version, raising concerns about data quality. 14 Our findings suggest that the self-administered version is appropriate for use by both patients and healthcare professionals; however, it should not replace the traditional version administered by trained evaluators, especially in clinical trials, due to the potential for scoring discrepancies.

Our results are consistent with the findings of Erb et al., 4 who also reported excellent agreement between the traditional in-person ALSFRS-R and its digital version, completed by both ALS patients and healthy controls. These findings support the potential of the self-administered version as a viable alternative for monitoring functional changes throughout disease progression. 4 Similarly, Maier et al. 7 emphasized that the online version of the ALSFRS-R can complement in-person visits, facilitating a more personalized and patient-centered approach that extends beyond standard follow-up schedules. 7

Furthermore, Mehdipour et al. concluded that the self-administered ALSFRS-R is cost-effective and psychometrically robust based on Rasch analysis, supporting its use for functional assessment in clinical and research settings. 1 Similarly, Montes et al. demonstrated that the self-administered ALSFRS-R is effective for monitoring disease progression at home. 13 These findings reinforce the relevance of our results and further support the reliability and applicability of the self-administered version for home-based monitoring.

Our study also builds upon the work of Guedes et al., 14 who validated the Portuguese version of the ALSFRS-R and confirmed its reproducibility and validity among Brazilian ALS patients. Their findings established the scale as a valuable tool for tracking symptom progression and limitations in daily activities. The present study extends this evidence by demonstrating that the self-administered version maintains this reliability and can be effectively applied within a Brazilian cohort.

The Bland-Altman analysis demonstrated narrow limits of agreement across all comparisons, supporting the consistency and robustness of the self-administered ALSFRS-R. These findings suggest that the self-administered version may serve as a reliable alternative for tracking ALS progression, particularly when in-person evaluations are not feasible. Nonetheless, any scores discrepancies should be interpreted with caution, especially in clinical trials where precision is paramount.

Therefore, this study provides solid evidence supporting the validity of the self-administered ALSFRS-R. Its implementation can enhance patient monitoring while reducing the logistical burdens associated with frequent in-clinic visits.

A key strength of this study is the validation of a format that allows patients to independently complete and submit the ALSFRS-R to researchers, without the need for a trained intermediary. This highlights the importance of remote monitoring and the potential to personalize the frequency of in-person consultations.

This approach also enables a more efficient use of consultation time, which is particularly valuable in ALS care, where clinical assessments are multifactorial and often complex, requiring comprehensive neurological evaluation.

The ability to track disease progression through self-assessment helps preserve relevant clinical information over time, facilitates the generation of individual reports, and supports data collection for analyzing progression rates in larger ALS cohorts. By empowering patients and enabling continuous data acquisition, this approach contributes to a more proactive, patient-centered, and data-driven model of care.

Nevertheless, further research is warranted to confirm its effectiveness in larger and more diverse ALS populations, as well as to explore its applicability across different healthcare settings.

In conclusion, the assessments conducted by both patients and researchers fell within the limits of agreement, indicating clinical feasibility. The self-administered version of the ALSFRS-R demonstrated reliability and suitability for remote monitoring of ALS patients. Future studies should investigate its implementation in various clinical settings to further validate its efficacy and effectiveness.
